# The importance of visual acuity screening in dental education amongst undergraduate dental students: a straightforward method

**DOI:** 10.3389/fdmed.2023.1337909

**Published:** 2024-01-10

**Authors:** Jaya Pindoria, Stefan Abela, Matthew Maguire, Martyn Sherriff, Dirk Bister

**Affiliations:** ^1^ King's College Dental Institute, London, United Kingdom; ^2^ Health Education England East of England, Cambridge, United Kingdom; ^3^ Ophthalmology Department, Guy's and St Thomas' Hospital NHS Foundation Trust, London, United Kingdom; ^4^ The University of Bristol Dental Hospital, Bristol, United Kingdom

**Keywords:** dental undergraduate, dental student, visual acuity, haptic training, virtual reality simulator, visual-motor skills

## Abstract

**Background:**

Visual acuity plays a pivotal role in a dental professional's daily performance and screening the students' field of vision in their early formative years ensures successful undergraduate programmes.

**Aims:**

To compare near and distance visual acuity and stereopsis in first-year and final-year dental students and investigate students' perception of their vision.

**Method:**

This was a cross-sectional study involving 100 KCL first- and final-year dental students who underwent assessment of their vision and completed a self-perception questionnaire. Near visual acuity was assessed using a standardised near vision test chart, distance visual acuity using COMPlog (Clinical Vision Measurement Systems Ltd, London, UK) computerised software and stereopsis using the Frisby Stereotest. Based on the Mann–Whitney test, no statistical differences were found between the first-year and final-year students’ near and distance visual acuity, nor in stereopsis difference at a significance level of *α* = 0.05. The null hypothesis was accepted.

**Results:**

84% of first-year students and 94% of final-year students attained the highest binocular near visual acuity score of 0.50M. Distance visual acuity scores showed a median ETDRS (Early Treatment Diabetic Retinopathy Study) Letters score 94 in the first-year group and 95 in the final-year group. 8% of students were found to have correctable refractive errors in distance visual acuity. The majority of students across both year groups were able to discern 20 s arc of the smallest disparity. The final-year students reported worrying about their eyesight significantly more than the first-year students.

**Conclusions:**

No statistically significant differences were found in near and distance visual acuity, and stereopsis, between first-year and final-year dental students. However, 8% of students were identified with undiagnosed, correctable refractive errors. The importance of students' vision in clinical dentistry is highlighted, and regular eye examination is recommended.

## 1 Introduction

The dental profession, in general, requires the acquisition of numerous skills including good patient management, good communication and very precise manual dexterity, amongst others. A fundamental skill required for any dental professional is the ability to carry out optimal clinical work by having unimpaired vision. Dental surgeons are required to accurately distinguish shapes, dimensions, colour, distances and depth. Motor skills are often tested on entry to dental school; however, currently there is no established requirement or assessment of dental students' vision on admission nor during their training programme. This research is particularly valuable as virtual reality simulators are becoming increasingly popular in dental education (1), and where visual acuity is key to having successful training outcomes.

The faculty of sight is very complex, and its different attributes include, but not limited to, colour vision, visual acuity and binocular vision. Concurrently, many factors influence visibility including illumination, distance and angle of object viewed, and size of object. High hand-eye coordination requires good visual acuity as well as other psychological and neurological qualities such as stereopsis (2).

Visual acuity is a measure of the ability of an eye to distinguish shapes and details of objects at a given distance (clarity and sharpness of vision). Dentistry, with its small operating field, requires visual control of small structures which is also known to decrease throughout life (3). Stereopsis can be defined as “the information regarding three-dimensional object structure which is made available through retinal image differences” (4). These differences arise because the eyes are horizontally separated by approximately 6 cm in humans. Previous studies have shown the following: (i) horizontal disparities can provide information about the slant, curvature and depth of proximally fixated objects (5–7); (ii) humans use this information (8–10); and (iii) the use of stereopsis (and other “cues”) is task dependent (11, 12).

Studies have repeatedly shown that dentists' self-assessment of their visual performance is unreliable. Responses to the questionnaires used in previous studies have shown a poor correlation with objective findings of the visual tests, with dental surgeons not being aware of their visual deficiencies (13, 14).

The current body of evidence looking at visual acuity and stereopsis in dental students is very limited. Most studies have small sample sizes and/or incorporate inherent biases. One study showed that dental students exhibited difficulty estimating depths and distances early on in their programme and that visual accuracy increased with clinical experience (15). Another study adopted a successful method of screening dental students for visual defects including squints, limits of convergence, and defective stereopsis (16). In a similar study a simple eyesight screening method led to identification of defective colour vision with implications on shade taking during prosthetic treatments (17). The aim of this study was to test the association between visual training effects and dental undergraduate training. The justification was obtained as there has been no testing and no results have been published testing this.

## 2 Aims

The aim of this cross-sectional study was to compare near and distance visual acuity and stereopsis in first-year and final-year dental students. The secondary aim was to investigate first-year and final-year undergraduate dental students' perception of their vision.

## 3 Materials and methods

### 3.1 Study participants

This was a cross-sectional observational study comparing first-year and final-year dental students at King's College London University, London, United Kingdom. All voluntary participants were recruited consecutively by the principal researcher (JP) between September 2018 and February 2019. A sample size of 47 was calculated using G*Power 3.1.9.7, Universität Düsseldorf, Düsseldorf, Germany for a significance *ɑ* = 0.05, power = 0.8 and a medium effect size = 0.6. To allow for drop-outs 50 students were recruited in each year. students were obtained in each of the year groups. The criteria for inclusion in the study were:

▪ Dental students at King’s College London University, London, United Kingdom, in the first year and final year of the dental undergraduate programme
▪ Above 18 years of age
▪ No previous eye surgery

### 3.2 Method

Participants were given an information sheet, a written consent form to complete, and a self-perception visual functioning questionnaire (Supplementary Material S1). A data capture form was used by the researchers to record anonymised participant demographics, date of birth, and gender and verify whether visual aids were worn at the time of the study. In cases where students wore glasses or contact lenses in everyday tasks, they were asked to wear these for the visual tests, thereby testing corrected vision. The same form was also used to record the findings from visual tests.

#### 3.2.1 Near visual acuity

**Figure 1:**
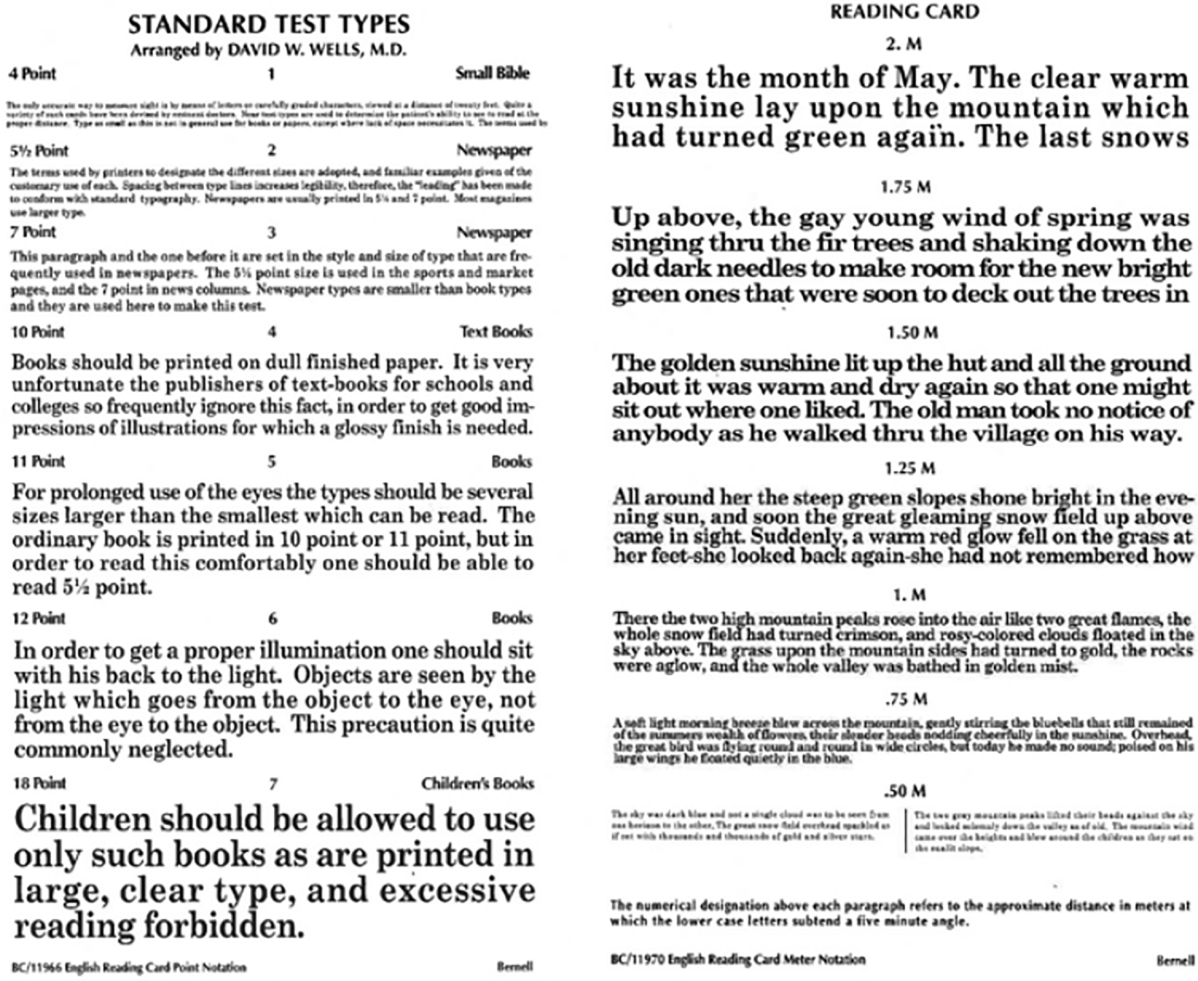
Near vision test chart, standard test types.

To assess near visual acuity, a standardised near vision test chart approved by the Faculty of Ophthalmologists was used (Figure 1). This test was carried out by one trained assessor for all participants for accuracy and repeatability. The reading chart uses Times New Roman font with sentences at varying sizes of print with logarithmic progression. The smallest print size that each student could read with fluency was recorded for each eye independently, right and then left, with the contralateral eye completely obscured using an occluder. The test was then repeated for binocular vision, both eyes unobscured. The test distance was standardised and measured to 40 cm in each case.

#### 3.2.2 Distance visual acuity

**Figure 2:**
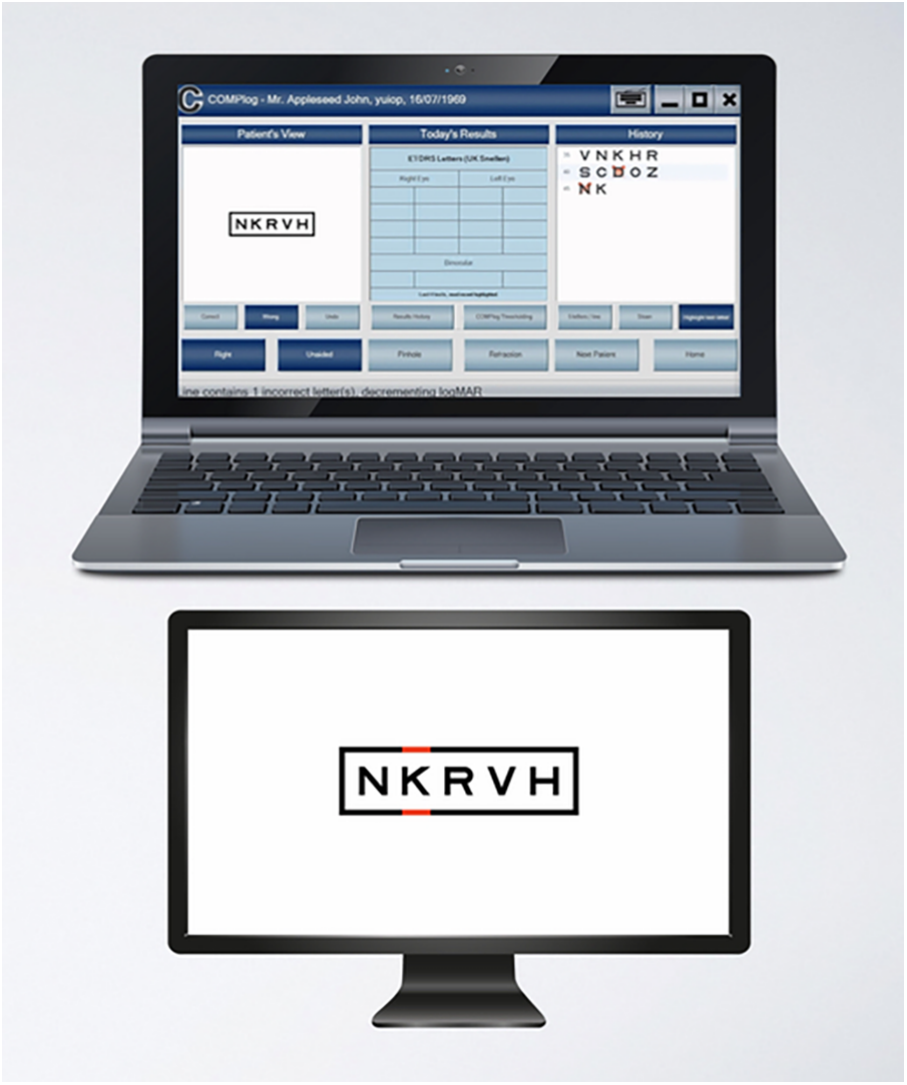
COMPlog distance visual acuity measurement system (14). Control monitor (top) and secondary monitor (bottom).

**Figure 3:**
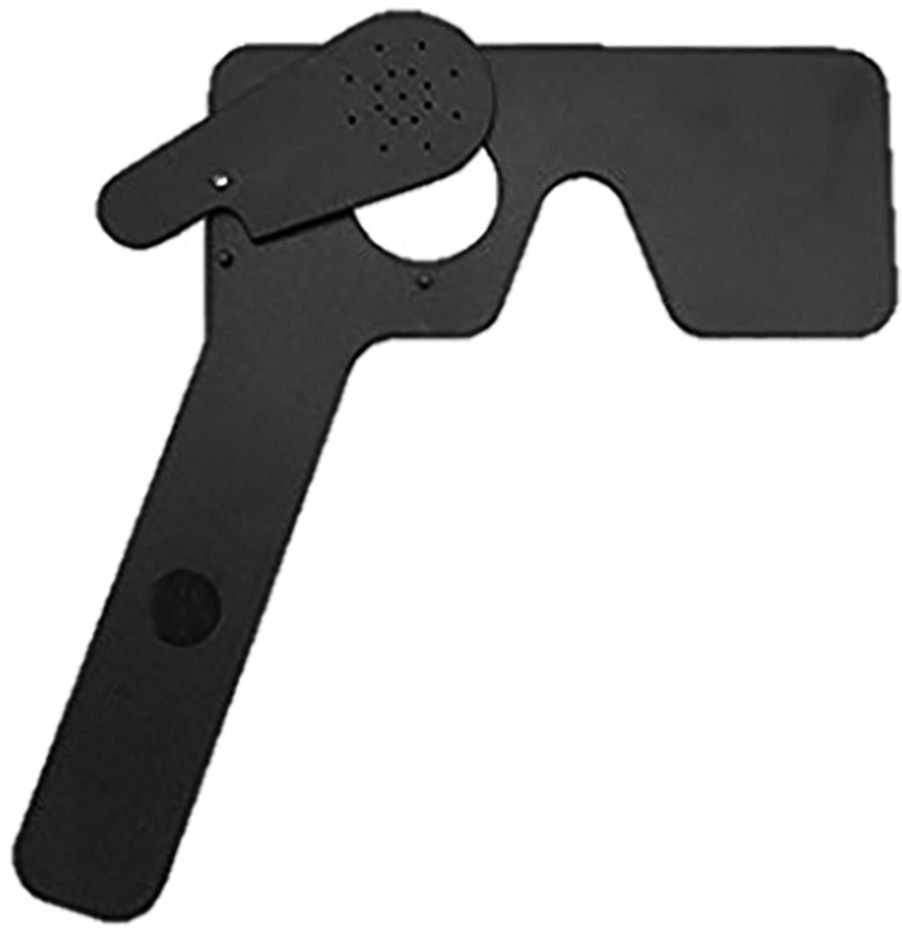
Pinhole occlude.

Distance visual acuity was evaluated using COMPlog (Complog Clinical Vision Measurement Systems Ltd., London, UK) computerised software (18). The software program was run on a laptop PC (control monitor) with a 24-inch widescreen secondary monitor (Figure 2). One assessor carried out all the assessments at a set distance of 3 m under consistent lighting conditions. When a participant is unable to identify all five letters on a row, the test is terminated, and the visual acuity is recorded in ETDRS (Early Treatment Diabetic Retinopathy Study) letters. Each eye was assessed individually, right and then left, with the contralateral eye completely obscured using an occluder. Each eye, right and left, was then tested individually using a pinhole occluder (Figure 3). The pinhole test allows a single ray of light from a point on an object to pass through the centre and is a useful method of determining reduced visual acuity due to refractive error (19). That is, vision can be corrected with glasses or contact lenses. The test was then repeated for binocular vision, both eyes unobscured.

#### 3.2.3 Stereopsis

**Figure 4:**
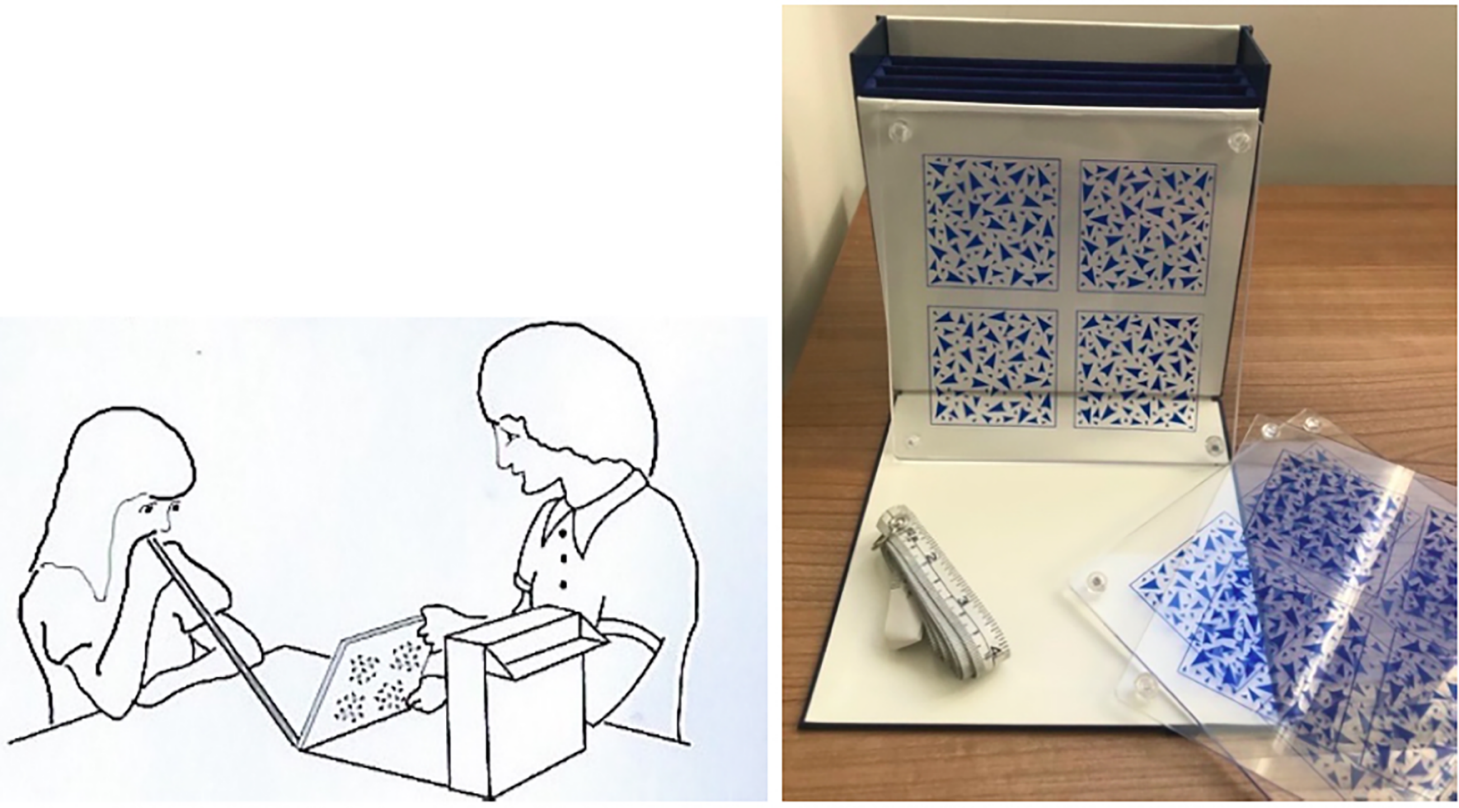
Diagram (left) of frisby stereotest and photo (right).

The Frisby Stereotest (Figure 4) was used to measure stereopsis which consists of three plates of varying thickness (6 mm, 3 mm, 1.5 mm). Each of the plates has four random texture patterns, with a hidden circular shape in one. With unobscured binocular vision, the student was asked to decide in which pattern the hidden shape lay. The stereotests were performed in random sequence in order to control for fatigue and learning effects. Using a tape measure, the viewing distance was increased incrementally per plate thickness to give the stereo threshold in units of seconds of arc. The lowest disparity that the student could reliably discriminate was calculated using Table 1 and recorded on the data capture form. All stereopsis tests were carried out by co-researcher MM who is trained in carrying out these tests.

**Table 1:** Disparities for stereoacuity assessment.

Viewing Distance Cm	Plate thickness		
	6 mm	3 mm	1.5 mm
30	600	300	150
40	340	170	85
50	215	110	55
60	150	75	40
70	110	55	31
80	85	40	21
100	55	25	15
120	40	20	10
150	25	10	5

#### 3.2.4 Self-perception questionnaire

Each student was asked to complete a shortened and adapted version of the self-perception visual functioning questionnaire (20), comprising a total of 18 questions in two domains; general health and difficulty with daily activities. The questions assessed the students' perceived views about their eyesight and difficulties encountered with daily activities as well as an additional question about accurately in a dental task of drilling a 1 mm cavity. The questionnaire used a Likert scale ranging from none of the time, 1; a little of the time, 2; some of the time, 3; most of the time, 4; and all of the time, 5. For the daily activity questions, the scale ranges from no difficulty at all, 1; a little difficulty, 2; moderate difficulty, 3; extreme difficulty, 4; stopped doing this because of your eyesight, 5; stopped doing this for other reasons or not interested in doing this, 6. As different scales were used, a composite score calculation could not be used, and each answer was to be assessed individually.

#### 3.2.5 Statistical Analysis

All the data analysis was conducted, analysed and processed using Stata Software, version 17, StataCorp, Texas, USA. The null hypothesis was that there is no statistically significant difference in near and distance visual acuity and stereopsis between first and final-year dental students. All statistical significant differences for near, distance visual acuity and stereopsis for the two cohorts were assessed using the Mann–Whitney test at a significance level of *α *= 0.05.

Responses from the questionnaire were analysed using an ordered logistic regression model when the data were ordered, and with a multinomial logistic regression analysis model where the responses had more than two discrete answers and were non-ordered. The reporting of this study conformed to the STROBE statement (Supplementary Material S2) (21).

## 4 Results

### 4.1 Sample characteristics

Table 2 illustrates the participant demographics with regard to gender, age, and use of visual aids.

**Table 2:** Sample demographics.

Year Group	1	5	Total
Gender (*n*)			
Female	30	34	64
Male	20	16	36
Age (years)			
Mean (sd)	19.3 (1.88)	23.6 (1.47)	
Max	25	27	
Min	18	22	
Visual aids (*n*)			
Unaided	30	26	56
Glasses	13	20	33
Contact lenses	7	4	11

### 4.2 Visual test results

#### 4.2.1 Near visual acuity

**Figure 5:**
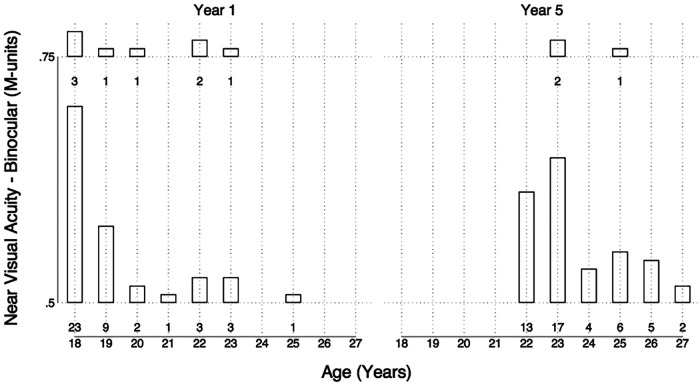
Bar chart of age plotted against binocular near visual acuity for year 1 students (left) and year 5 students (right).

The majority of dental students in year 1 (84%, *n* = 42) and in year 5 (94%, *n* = 47) had a binocular near visual acuity score of 0.50 M, the highest attainable score using our methods (Figure 5). Eight first year students scored 0.75 M whereas three final-year students scored 0.75 M. Ordered logistic regression showed no statistically significant differences between the year groups in binocular near visual acuity. This was also true when the right eye and left eye were tested independently. There was no correlation found between age and near visual acuity.

#### 4.2.2 Distance visual acuity

**Figure 6:**
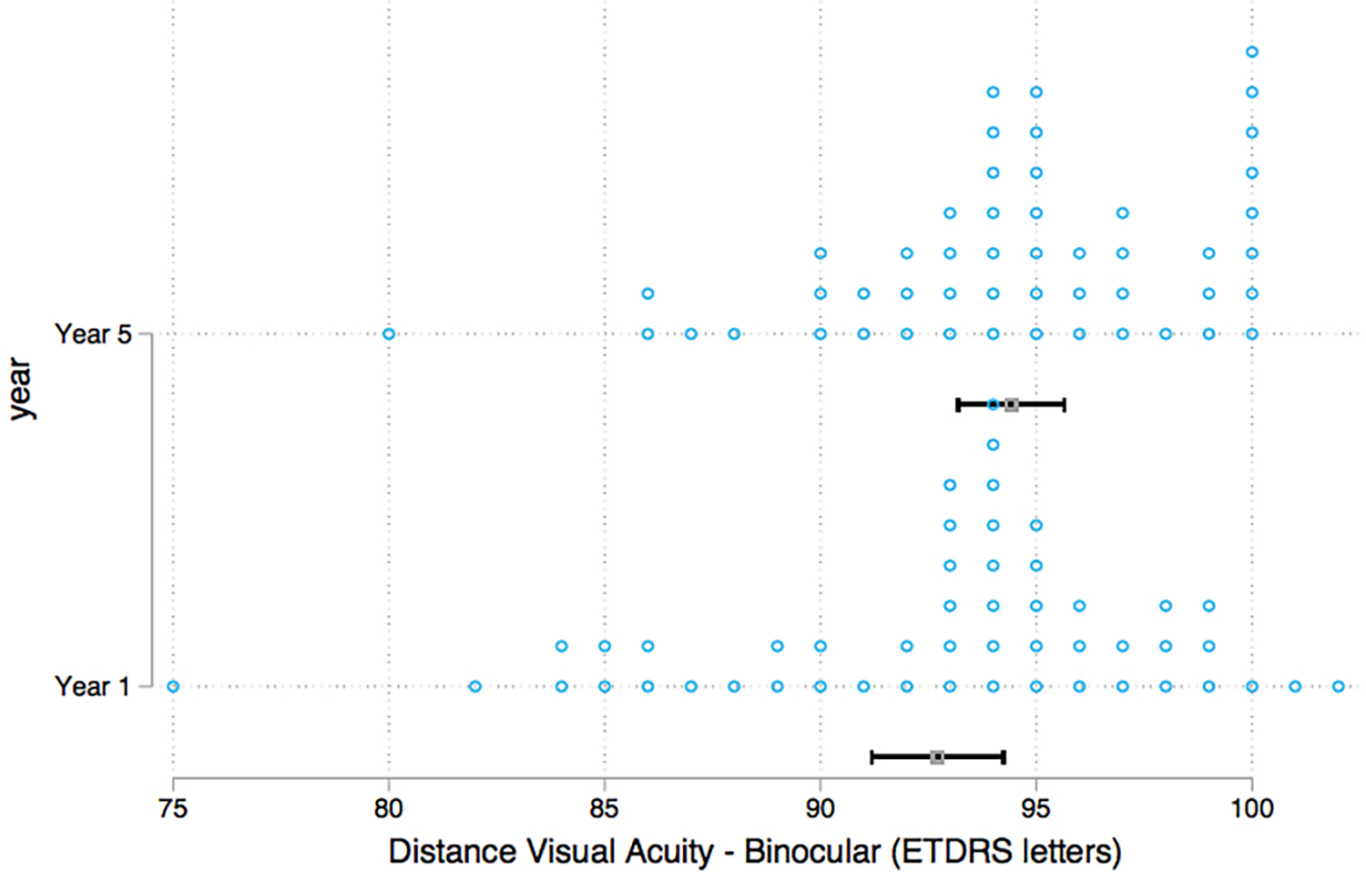
Strip plot of binocular distance visual acuity per year group.

**Figure 7:**
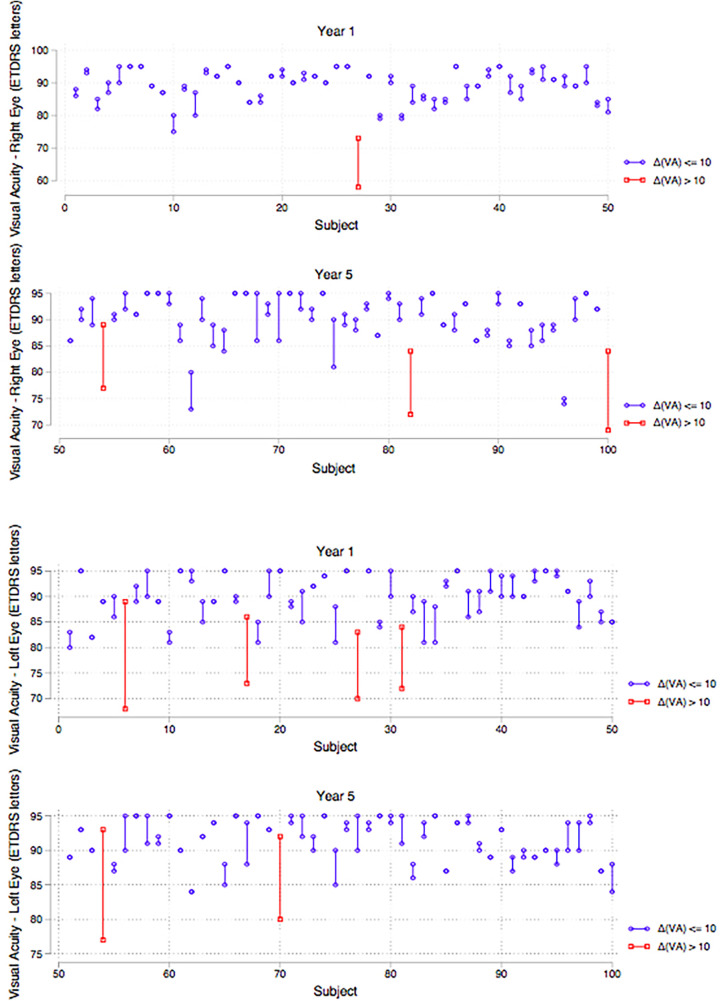
Strip plots of distance visual acuity per year group showing students with improvement in distance visual acuity of >10 ETDRS letters with the use of the pinhole occluder (highlighted). Top (right eye), bottom (left eye).

Binocular distance visual acuity was similar between groups with a median score of 94 ETDRS in the first-year group and 95 ETDRS letters in the final-year group (Figure 6). Mann–Whitney test was used to test for differences between the year groups, and the results showed no statistically significant differences in binocular distance visual acuity. A closer evaluation of individual participants revealed that eight students (*n* = 4 year 1, *n* = 4 year 5) displayed a clinically significant improvement in distance visual acuity of more than 10 ETDRS letters with the use of the pinhole occluder (Figure 7).

#### 4.2.3 Stereopsis

**Figure 8:**
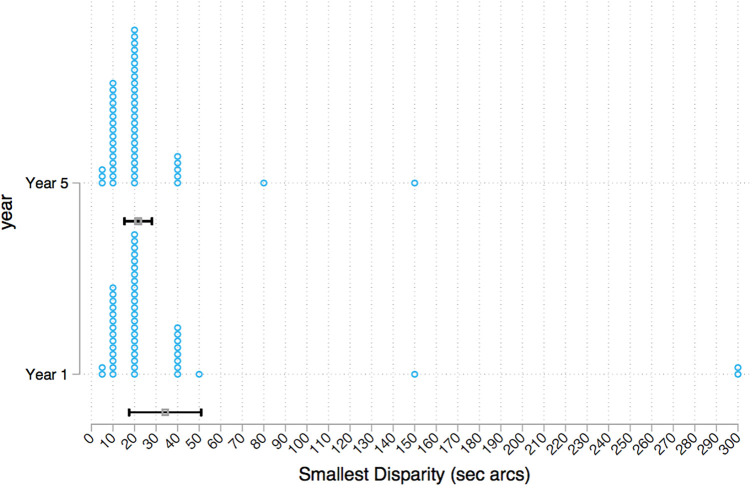
Strip plot of smallest disparity per year group.

A comparison of the smallest disparity that the student could reliably discriminate found no significant difference between first-year and final-year students when assessed using the Mann–Whitney test. The majority of students in both year groups scored 20 s arcs. In the first-year group, 44% (*n* = 22) of students achieved this score and 48% (*n* = 24) in the final-year group (Figure 8).

### 4.3 Self-perception questionnaire results

There was a statistically significant difference in the groups when asked “How much of the time do you worry about your eyesight?” (*p* = 0.044) with the final-year group worrying more about their eyesight compared to the first-year group.

## 5 Discussion

The responsibility of good visual health is currently reliant on the individual dental student undertaking independent eye examinations and is by no means essential. From a safety standpoint, when compared to other professions, admission criteria pertaining to visual ability is less stringent in dentistry. In the UK aviation industry, for example, eye examinations are a mandatory requirement for medical certification, revalidation and renewal of aircrew.

Corrected vision was tested in students who were frequent glasses or contact lens users, thus allowing visual testing vision in an everyday setting. The lighting in the room used for the visual tests was kept at a constant; however, in the dental setting an operating light offers more illumination than standard room lighting. The typical working distance of a dentist is 300 mm, and whilst the near acuity reading tests were carried out at a range similar to this, the remaining tests were not due to the design of the tests.

All students had a binocular near visual acuity of at least 0.75 M with the majority obtaining 0.50 M (84% in the first year, 94% in the final year), the highest attainable near visual acuity score with the test type used. Although not statistically significant, this is a noteworthy 10% difference of clinical relevance indicating 1 in 10 final-year students with better near visual acuity compared to first year students. Eight first year students scored 0.75 M whereas only three final-year students scored 0.75 M, the remainder scoring 0.50 M. This difference was not statistically significant but illustrated that a greater number of students with better near visual acuity were in the final-year cohort. This trend would be congruous with the findings of Forgie et al. (22) who assessed 46 practising dentists and found them all to have 0.50 M near visual acuity, using a standardised reading type test. A limitation of the test type used was its sensitivity as most students could read the smallest typeface used.

This study found that distance visual acuity was not statistically significantly different between the groups. However, undiagnosed refractive errors were highlighted by the use of the pinhole occluder which improved distance acuity by 10 ETDRS letters in 4 students in each group. Undiagnosed refractive errors in working age UK population have been reported at a 1.6% prevalence (23); however, this figure was derived from a disparate cohort aged 44/45 years. 8% of dental students with a potential benefit from wearing lenses is a sizeable proportion in a profession that relies heavily on perceptual-motor skills and where optimal vision is essential for patient safety. These students with correctable refractive errors were advised to seek further independent eye tests. Ignoring this may impact on students' clinical progression and also raises issues pertaining to admission criteria. Currently, occupational health clearance is required to enter dental training within the UK, and the authors propose that evidence of a recent eye test be also incorporated into entry requirements as well as regular testing throughout the dental undergraduate training.

The findings of the present study were not in line with a previously conducted study by Dimitrijevic et al*.* (15) who found that entry-level dental students performed significantly worse in depth perception and estimation than senior dental students. In contrast, the results showed that there was no significant difference between the year groups in the smallest disparity of stereopsis. Overall, stereopsis was good for both year groups with both having median and mode results at 20 s arcs. This is in accordance with Bohr and Read (24), who reported median stereoacuity on the Frisby test at 20 s arcs, when assessing a large sample aged 11–49 years old.

The majority of students regarded their eyesight as “very good” or “excellent”. There was no statistical difference between self-reported quality of eyesight between the two year groups. Eight students were found to have undiagnosed refractive errors whilst seven reported their eyesight quality as “good”/“very good”/“excellent”. The results of this study add to the existing literature that dentist's self-assessment of their vision is unreliable.

When asked how much of the time students worry about their eyesight, there was a significant difference between the year groups, with first-year students reporting worrying less of the time than final-year students (*p* = 0.044). Interestingly this discrepancy in concern over eyesight is not supported by objective test findings. It is possible that with more clinical experience, dental students are conscious of the need for appropriate optical correction. Although there is a large difference in clinical experience, there was no statistical difference between the year groups when asked about accuracy in drilling a 1 mm cavity, with most stating they could complete this task with no difficulty at all.

The use of questionnaires in cross-sectional studies to analyse students' perceptions is a widely used method and has been used by all dental specialties (25). The limitations of evaluating self-perception of vision in this study were mainly based on the reliance on the participants' truthfulness in completing the questionnaire and the incorporation of subjective bias. Albeit assured anonymity, the students may overestimate their visual capabilities in fear of a detrimental effect on their academic or clinical progression.

## 6 Conclusions

This study did not show any statistically significant differences between the year groups in near visual acuity and distance visual acuity, nor in stereopsis. Therefore, the null hypothesis could not be rejected and has been accepted. Eight percent of students had undiagnosed, correctable refractive errors with distance visual acuity. Students with visual deficits should be alerted and supported to undergo further eye examinations throughout their training programme.

Dental students' self-assessment of their vision is unreliable, and therefore routine testing of all dental students for perceptual and visual difficulties is recommended. This study has a wide range of purpose with the findings being potentially applicable to most universities providing dental undergraduate programmes. Students, as well as practising dentists, should be aware of the importance of their vision in clinical dentistry, and regular attendance for an eye examination is to be encouraged and recommended. Screening for visual acuity and stereopsis is easily implemented, and it is hard to justify overlooking a potential impairment to clinical dentistry.

## 7 Future research

This study was a cross-sectional study with measurements of two different cohorts taken at one point in time. A future prospective longitudinal study design would be more accurate in assessing changes in vision throughout dental training. The influence of loupes on visual acuity and stereopsis and their effect on compensation on visual deficits could be a very useful study as loupes are being increasingly used by dental professionals.

There is also a need for an investigation into the effect of students' vision on educational performance, and whether compensatory learning can occur in the presence of visual impediments. The interaction of visual acuity and stereopsis on operative procedures of varying specialties within dentistry also remains the subject of further studies.

As virtual reality and augmented realities are becoming more commonplace in dental undergraduate programmes, with potential implications on visual acuity and stereopsis, increased trials, investigations and validation of these systems will be crucial in delivering effective dental programmes (26).

## Data Availability

The original contributions presented in the study are included in the article/Supplementary Material, further inquiries can be directed to the corresponding author.

## References

[1] Towers A Field J Stokes C Maddock S Martin N. A scoping review of the use and application of virtual reality in pre-clinical dental education. Br Dent J. (2019) 226:358–66. 10.1038/s41415-019-0041-0 30850794

[2] Syrimi M Ali N. The role of stereopsis (three-dimensional vision) in dentistry: review of the current literature. Br Dent J. (2015) 218(10):597–8. 10.1038/sj.bdj.2015.387 25998354

[3] Eichenberger M Perrin P Neuhaus KW Bringolf U Lussi A. Visual acuity of dentists under simulated clinical conditions. Clin Oral Investig. (2013) 17(3):725–9. 10.1007/s00784-012-0753-x 22638771 PMC3627031

[4] Mon-Williams M Mushtaq F Wilkie RM Khambay B Manogue M. A three dimensional view of stereopsis in dentistry. Br Dent J. (2015) 219:26–7. 10.1038/sj.bdj.2015.881 26611301

[5] Stevens KA Brookes A. Integrating stereopsis with monocular interpretations of planar surfaces. Vision Res. (1988) 28:371–86. 10.1016/0042-6989(88)90180-0 3188401

[6] Banks MS Hooge IT Backus BT. Perceiving slant about a horizontal axis from stereopsis. J Vis. (2001) 1:55–79. 10.1167/1.2.1 12678602

[7] Wickens CD Merwin DH Lin EL. Implications of graphics enhancements for the visualization of scientific data: dimensional integrality, stereopsis, motion, and mesh. Hum Factors. (1994) 36:44–61. 10.1177/001872089403600103 8026843

[8] Fielder AR Moseley MJ. Does stereopsis matter in humans? Eye (Lond). (1996) 10:233–8. 10.1038/eye.1996.51 8776453

[9] Saladin JJ. Stereopsis from a performance perspective. Optom Vis Sci. (2005) 82:186–205. 10.1097/01.OPX.0000156320.71949.9D 15767874

[10] Schreiber K Crawford JD Fetter M Tweed D. The motor side of depth vision. Nature. (2001) 410:819–22. 10.1038/35071081 11298450

[11] Read JCA Begum SF McDonald A Trowbridge J. The binocular advantage in visuomotor tasks involving tools. Iperception. (2013) 4:101–10. 10.1068/i0565 23755355 PMC3677330

[12] Greenwald HS Knill DC. A comparison of visuomotor cue integration strategies for object placement and prehension. Vis Neurosci. (2008) 26:63–72. 10.1017/S0952523808080668 18759994 PMC2943639

[13] Eichenberger M Perrin P Ramseyer ST Lussi A. Visual acuity and experience with magnification devices in Swiss dental practices. Oper Dent. (2015) 40:E142–9. 10.2341/14-103-C 25748209

[14] Perrin P Eichenberger M Neuhaus MW Lussi A. A near visual acuity test for dentists. Oper Dent. (2017) 42:581–6. 10.2341/16-128-L 28708006

[15] Dimitrijevic T Kahler B Evans G Collins M Moule A. Depth and distance perception of dentists and dental students. Oper Dent. (2011) 36:467–77. 10.2341/10-290-L 21859316

[16] Rawlinson A. A study to investigate the visual quality of dental undergraduates using a simple screening programme. Aust Dent J. (1988) 33:303–7. 10.1111/j.1834-7819.1988.tb04182.x 3252782

[17] Rawlinson A. A simple eyesight screening programme for dental undergraduates: results after 7 years. Aust Dent J. (1993) 38:394–9. 10.1111/j.1834-7819.1993.tb05522.x 8259917

[18] COMPlog. 2012. Online information available at: http://complog-visual-acuity.com/ (Accessed 17 January 2020).

[19] Elkington AR Frank HJ Greaney M J. Clinical optics. 3rd ed Malden, Mass: John Wiley and Sons Inc. (1999).

[20] Mangione CM Lee PP Gutierrez PR Spritzer K Berry S Hays RD. Development of the 25-item national eye institute visual function questionnaire (VFQ-25). Arch Ophthalmol. (2001) 119:1050–8. 10.1001/archopht.119.7.1050 11448327

[21] von Elm E Altman DG Egger M Pocock SJ Gøtzsche PC Vandenbroucke JP Strengthening the reporting of observational studies in epidemiology (STROBE) statement: guidelines for reporting observational studies. Br Med J. (2007) 335(7624):806–8. 10.1136/bmj.39335.541782.AD 17947786 PMC2034723

[22] Forgie AH Gearie T Pine CM Pitts NB. Visual standards in a sample of dentists working within Scotland. Prim Dent Care. (2001) 8:124–7. 10.1308/135576101322561976 11490703

[23] Rahi JS Peckham CS Cumberland PM. Visual impairment due to undiagnosed refractive error in working age adults in Britain. Br J Ophthalmol. (2008) 92:1190–4. 10.1136/bjo.2007.133454 18723742

[24] Bohr I Read JCA. Stereoacuity with frisby and revised FD2 stereo tests. Public Lib of Sci One. (2013) 8:1–12. 10.1371/journal.pone.0082999 24349416 PMC3861460

[25] Lone MA Iqbal UA Lone MM Adnan S Heboyan A Ahmed N Current trends in fixed prosthodontics education in undergraduate dental colleges. J Med Educ Curric Dev. (2023) 10:23821205231193282. 10.1177/23821205231193282 37614332 PMC10443680

[26] Dzyuba N Jandu J Yates J Kushnerev E. Virtual and augmented reality in dental education: the good, the bad and the better. Eur J Dent Educ. (2022) 00:1–19. 10.1111/eje.12871 36336847 PMC12287989

