# Supplementary material for: The importance of visual acuity screening in dental education amongst undergraduate dental students: a straightforward method
Source: Front Dent Med. 2024 Jan 10;4:1337909. doi: 10.3389/fdmed.2023.1337909 (PMC11797794; doi:10.3389/fdmed.2023.1337909)
Supplement: Supplementary file 1 [file Datasheet1.pdf]

Supplementary material

Self-perception Visual Functioning Questionnaire

Self-Perception
Visual Functioning Questionnaire v1 02/06/18

The following is a survey with statements about problems which involve your vision or feelings that you have about your vision condition. After each question please choose the response that best describes your situation.

Please answer all the questions as if you were wearing your glasses or contact lenses (if any).

Please take as much time as you need to answer each question. All your answers are confidential. In order for this survey to improve our knowledge about vision problems and how they affect your quality of life, your answers must be as accurate as possible.

INSTRUCTIONS:

1. In general we would like to have people try to complete these forms on their own. If you find that you need assistance, please feel free to ask the project staff and they will assist you.

2. Please answer every question.

3. Answer the questions by circling the appropriate number.

4. If you are unsure of how to answer a question, please give the best answer you can and make a comment in the left margin.

5. Please complete the questionnaire before leaving and give it to a member of the project staff. Do not take it home.

6. If you have any questions, please feel free to ask a member of the project staff, and they will be glad to help you.

STATEMENT OF CONFIDENTIALITY:

All information that would permit identification of any person who completed this questionnaire will be regarded as strictly confidential. Such information will be used only for the purposes of this study and will not be disclosed or released for any other purposes without prior consent, except as required by law.

This survey has been adapted from the "National Eye Institute Visual Functioning Questionnaire 25" (VFQ-25) developed at RAND, July 1996, under the sponsorship of the National Eye Institute.

PART 1 - GENERAL HEALTH AND VISION

1. In <u>general</u>, would you say your overall <u>health</u> is:

(Circle One)

Excellent 1
Very Good 2
Good 3
Fair 4
Poor 5

2. At the present time, would you say your eyesight using both eyes (with glasses or contact lenses, if you wear them) is excellent, good, fair, poor, or very poor or are you completely blind?

(Circle One)

Excellent 1
Very Good 2
Good 3
Fair 4
Poor ……............................. 5

3. How much of the time do you worry about your eyesight?

(Circle One)

None of the time 1
A little of the time 2
Some of the time 3
Most of the time 4
All of the time? 5

PART 2 - DIFFICULTY WITH ACTIVITIES

The next questions are about how much difficulty, if any, you have doing certain activities wearing your glasses or contact lenses if you use them for that activity.

4. How much difficulty do you have reading ordinary print in newspapers? Would you say you have:

(Circle One)

No difficulty at all 1
A little difficulty 2
Moderate difficulty 3
Extreme difficulty 4
Stopped doing this because of your eyesight 5
Stopped doing this for other reasons or not interested in doing this 6

5. How much difficulty do you have doing work or hobbies that require you to see well up close, such as cooking, sewing, fixing things around the house, or using hand tools? Would you say:

(Circle One)

No difficulty at all 1
A little difficulty 2
Moderate difficulty 3
Extreme difficulty 4

Stopped doing this because of your eyesight 5
Stopped doing this for other reasons or not
interested in doing this 6

6. Wearing glasses, how much difficulty do you have reading the small print in a telephone book, on a medicine bottle, or on legal forms?
Would you say:

(Circle One)

No difficulty at all 1
A little difficulty 2
Moderate difficulty 3
Extreme difficulty 4
Stopped doing this because of your eyesight 5
Stopped doing this for other reasons or not
interested in doing this 6

7. Because of your eyesight, how much difficulty do you have doing things like <u>shaving, styling your hair, or putting on makeup</u>?

(Circle One)

No difficulty at all 1
A little difficulty 2
Moderate difficulty 3
Extreme difficulty 4
Stopped doing this because of your eyesight 5
Stopped doing this for other reasons or not
interested in doing this 6

8. How <u>accurately</u> do you think you can <u>draw two points 1mm apart</u>?

(Circle One)

No difficulty at all 1
A little difficulty 2
Moderate difficulty 3
Extreme difficulty 4

9. Because of your eyesight, how much difficulty do you have finding something on a crowded shelf?

(Circle One)

No difficulty at all 1
A little difficulty 2
Moderate difficulty 3
Extreme difficulty 4
Stopped doing this because of your eyesight 5
Stopped doing this for other reasons or not
interested in doing this 6

10. How <u>accurately</u> do you think you can drill a <u>1mm</u> cavity into enamel?

(Circle One)

No difficulty at all 1
A little difficulty 2
Moderate difficulty 3
Extreme difficulty 4

11. Because of your eyesight, when pouring liquid, how much difficulty do you have judging the level of the liquid in a container? e.g. the level of coffee in a cup

(Circle One)

No difficulty at all 1
A little difficulty 2
Moderate difficulty 3
Extreme difficulty 4

12. When reaching for an object, how often do you find that it is further away or closer than you thought?

(Circle One)

None of the time 1
A little of the time 2
Some of the time 3
Most of the time 4
All of the time? 5

13. How often do you have problems judging how close or far things are from you?

(Circle One)

None of the time 1
A little of the time 2
Some of the time 3
Most of the time 4
All of the time? 5

14. How much difficulty do you have reading street signs or the names of stores?

(Circle One)

No difficulty at all 1
A little difficulty 2
Moderate difficulty 3
Extreme difficulty 4
Stopped doing this because of your eyesight 5
Stopped doing this for other reasons or not interested in doing this 6

15. Because of your eyesight, how much difficulty do you have recognising people you know from across a room?

(Circle One)

No difficulty at all 1
A little difficulty 2
Moderate difficulty 3
Extreme difficulty 4
Stopped doing this because of your eyesight 5
Stopped doing this for other reasons or not
interested in doing this 6

16. Because of your eyesight, how much difficulty do you have <u>taking part in active sports or other outdoor activities that you enjoy</u> (like golf, bowling, jogging, or walking)?

(Circle One)

No difficulty at all 1
A little difficulty 2
Moderate difficulty 3
Extreme difficulty 4
Stopped doing this because of your eyesight 5
Stopped doing this for other reasons or not
interested in doing this 6

17. Because of your eyesight, how much difficulty do you have <u>seeing and enjoying programs on TV</u>?

(Circle One)

No difficulty at all 1
A little difficulty 2
Moderate difficulty 3
Extreme difficulty 4
Stopped doing this because of your eyesight 5
Stopped doing this for other reasons or not
interested in doing this 6

18. Because of your eyesight, how much difficulty do you have <u>noticing objects off to the side while you are walking along</u>?

(Circle One)

No difficulty at all 1
A little difficulty 2
Moderate difficulty 3
Extreme difficulty 4
Stopped doing this because of your eyesight 5
Stopped doing this for other reasons or not
interested in doing this 6
